# S100s and HMGB1 Crosstalk in Pancreatic Cancer Tumors

**DOI:** 10.3390/biom13081175

**Published:** 2023-07-28

**Authors:** Angelo Mandarino, Swetha Thiyagarajan, Allana C. F. Martins, Roberto da Silva Gomes, Stefan W. Vetter, Estelle Leclerc

**Affiliations:** Department of Pharmaceutical Sciences, North Dakota State University, Fargo, ND 58105, USA

**Keywords:** S100 protein, HMGB1, pancreatic cancer

## Abstract

Pancreatic cancer remains a disease that is very difficult to treat. S100 proteins are small calcium binding proteins with diverse intra- and extracellular functions that modulate different aspects of tumorigenesis, including tumor growth and metastasis. High mobility group box 1 (HMGB1) protein is a multifaceted protein that also actively influences the development and progression of tumors. In this study, we investigate the possible correlations, at the transcript level, between S100s and HMGB1 in pancreatic cancer. For this purpose, we calculated Pearson’s correlations between the transcript levels of 13 cancer-related S100 genes and HMGB1 in a cDNA array containing 19 pancreatic cancer tumor samples, and in 8 human pancreatic cancer cell lines. Statistically significant positive correlations were found in 5.5% (5 out of 91) and 37.4% (34 of 91) of the possible S100/S100 or S100/HMGB1 pairs in cells and tumors, respectively. Our data suggest that many S100 proteins crosstalk in pancreatic tumors either with other members of the S100 family, or with HMGB1. These newly observed interdependencies may be used to further the characterization of pancreatic tumors based on S100 and HMGB1 transcription profiles.

## 1. Introduction

Pancreatic cancer is a devastating disease with a current 5-year survival rate of 12% [1]. The generation and development of pancreatic tumors is complex, and many actors participate in the process [2]. In recent years, S100 proteins and high mobility group box 1 (HMGB1) were shown to actively participate in multiple aspects of pancreatic cancer [3,4]. HMGB1 is a multifaceted protein with nuclear, cytoplasmic, and extracellular functions [5]. S100 proteins form a family of 20 structurally related EF-hand calcium binding proteins that possess both intracellular and extracellular functions [6,7,8]. Many members of the S100 protein family are upregulated in cancer tissues and participate in the growth of tumors and/or formation of metastases [7,9,10]. In pancreatic cancer, several S100 proteins have been found to be differently expressed between normal and cancerous pancreatic tissues (reviewed in [3]). Among these S100 proteins, S100P and S100A4 have been most intensively investigated and both proteins have been shown to stimulate pancreatic cancer cell proliferation and tumor growth [11,12,13,14,15,16,17]. However, the role of the other members of the S100 family has not been as thoroughly investigated. In this study, we investigated the presence of possible correlations, at the transcript level, between 13 S100 genes (S100A2, S100A4, S100A6, S100A7, S100A8, S100A9, S100A10, S100A11, S100A12, S100A13, S100A14, S100A16 and S100P) and HMGB, in a cDNA array of 19 pancreatic tumors, and in 8 well-characterized human cancer cell lines (AsPC-1, BxPC-3, Capan-2, CFPAC-1, HPAF-II, Hs 766T, MIA PaCa-2, and PANC-1). We found several differential expression patterns for S100 proteins in cell lines and tumors and observed changes in expression profiles between cell lines and tumors. Using Pearson’s correlation (R) analysis to estimate correlation strengths between S100s and HMGB1, we observed significantly more and stronger correlations within tumor samples than within cell lines. This suggests that the tumor environment influences the expression of S100 proteins and HMGB1. These newly observed interdependencies may be used to further the characterization of pancreatic tumors based on S100 and HMGB1 transcription profiles.

## 2. Materials and Methods

### 2.1. Cell Lines

The 8 pancreatic cancer cell lines were purchased from ATCC (Manas, VA) and grown in their recommended media containing 10% FBS and supplemented with penicillin (100 U/mL) and streptomycin (100 μg/mL). The respective media for each cell line was as follows: AsPC-1 (RPMI), BxPC-3 (RPMI), Capan-2 (McCoy’s 5a), CFPAC-1 (IMDM), HPAF-II (EMEM), Hs 766T (EMEM), MIA PaCa-2 (DMEM), and PANC-1 (DMEM). In addition, MIA PaCa-2 cell culture media was supplemented with 2% horse serum, as recommended by ATCC. These cell lines were established from tumors or ascites isolated from pancreatic cancer patients. Four cell lines were established from primary tumors (BxPC-3, Capan-2, MIA PaCa-2, and Panc-1), two from ascites (AsPC-1, HPAF-2), and two from metastatic tissues (CFPAC-1 and Hs766T). These cell lines have been characterized at the molecular level and histologically, and showed variations in histologic differentiation, as well as mutations in at least one of the following four carcinogenesis-driving genes: K-Ras, P53, P16, and SMAD4 (summarized in Table 1 from reference [18]).

Normal human pancreatic duct cells (hTERT-HPNE) stably transfected with human telomerase reverse transcriptase (hTERT) were from ATCC and used as non-cancerous control cells. These cells were grown in DMEM high glucose (D6429; Sigma-Aldrich, Saint Louis, MO, USA), supplemented with 10% fetal bovine serum, 10 ng/mL human EGF (SRP3027; Sigma-Aldrich) and 750 ng/mL puromycin (sc-108071A; Santa Cruz Biotechnology, Dallas, TX, USA).

### 2.2. Pancreatic Tumor cDNA Array

The pancreatic tumor cDNA PNRT101 arrays were purchased from OriGene Technologies (Rockville, MD, USA). The arrays consisted of 23 samples including 4 normal, 2 stage IB, 2 stage IIB, 2-III, and 13 samples where the stage of the tumor was not reported. As described in the product documentation, the array cDNAs were synthesized from high quality total RNAs of pathologist-verified tissues, and normalized with b-actin in two sequential qPCR analyses. For this reason, the Ct values obtained from each of the 19 tumor samples were used, without normalization with actin, for the correlation studies.

### 2.3. Real-Time PCR

For the preparation of RNAs from the cultured cell lines, cells were grown to 70–80% confluency and lysed with a lysis solution provided with the PARIS kit (Thermo Fisher Scientific, Waltham, MA, USA). RNAs were extracted and purified using the same kit and RNA concentrations were determined by UV absorption at 260 nm. The quality of the RNAs was assessed by gel electrophoresis on 1% agarose gels. RNAs (1 μg/20 μL sample) were reverse-transcribed into cDNA using the M-MuLV reverse transcriptase (New England Biolabs (Ipswich, MA, USA).

RT-PCR experiments were performed using 10 ng cDNA in 20 mL reaction volume using the HOT FIREPol^®®^ EvaGreen^®®^qPCR Mix (Solis Biodyne/Mango Biotechnology LLC, Mountain View, CA, USA) and 250 nM of each forward and reverse primer. For each RT-PCR, 40 cycles were run. Each cycle consisted of a 15 s dissociation step at 95 °C, followed by a 20 s annealing step at 60 °C, and a 20 s extension step at 72 °C. Dissociation plots for each sample were analyzed to ensure that specific products, with melting temperature (Tm) above 85 °C, were generated during the PCR. In addition, the presence of a PCR amplicon of the expected molecular size was assessed by agarose gel electrophoresis. The primers used to detect the transcripts of actin and of the various S100s are listed in Table 2.

For the RT-PCR performed with the cDNAs from cultured cells, the Ct values for each gene were obtained from the amplification plots, and the Cts were calculated using -actin as a housekeeping gene (Ct = Ct_gene_ − Ct_actin_). For each gene, at least three RT-PCRs were performed, using independent RNA preparations. The Cts were then used for statistical analysis. For the RT-PCR performed with the pancreatic tumor cDNA array, the Ct values for each gene and each array sample were also obtained from the amplification plots and used for the statistical analysis. RT-PCRs were determined on average two times for each set of primers with the tumor array. As described by Origene, the cDNA arrays contained 2–3 ng cDNA per well. Ct values obtained from the tumor array were used for statistical analysis.

### 2.4. Statistical Analysis

When analyzing the data generated from the cultured pancreatic cell lines, the mean Cts (Ct = Ct_gene_ − Ct_actin_), relative to the housekeeping gene β-actin, and their standard deviations were calculated. Low Cts corresponded to high levels of transcripts, and conversely, high Cts corresponded to low levels of transcripts relative to actin (Table 3). Pearson’s correlation coefficients (R and *p*-value) were calculated between S100s and HMGB1 transcripts using the Cts from the 8 cell lines (Table 4) using Microsoft Excel. The *p*-values corresponding to the Pearson correlation coefficients were calculated using the regression program provided by Microsoft Excel. When analyzing the data generated from the pancreatic tumor cDNA array, Pearson’s correlation coefficients of S100/S100 and S100/HMGB1 pairs were calculated directly from the Ct values because the cDNAs had been previously normalized and validated with b-actin in two sequential qPCR analyses by the provider (Origene) (Table 5). The Ct values from each of the 19 pancreatic tumor samples were used for the correlation studies. The *p*-values corresponding to the Pearson correlation coefficients were calculated using the regression program provided by Microsoft Excel (Table 5). Correlation strengths were described as very strong (R > 0.7), strong (0.5 < R < 0.7), or medium/moderate (0.4 < R < 0.5).

## 3. Results

Transcript levels for 13 S100 proteins and HMGB1, determined by qPCR, are summarized in Table 3 for cell lines. Correlation analysis results are shown in Table 4 for cell lines and Table 5 for tumor samples.

### 3.1. S100A2

In cancer cell lines, S100A2 presented the lowest transcript level among the 13 selected S100 genes. Indeed, the Ct values for S100A2 were above 16 for 6 cell lines. Hs766T and BxPc3 showed the highest transcript levels, with Ct of 11.45 +/− 0.65 and 9.17 +/− 1.78, respectively. Interestingly, these two cell lines are the only cell lines in our panel that have wild-type KRAS genotypes. Overall, the lowest level of S100A2 transcripts was observed in the non-cancerous human pancreatic epithelial HPNE cell line with Ct of 19.80 +/− 0.12 (Table 3).

Correlation analysis identified a very strong positive correlation (R = 0.82) between S100A2 and S100A13 in cancerous cell lines (Table 4), but that correlation was not observed in the tumor array.

In the tumor array, S100A2 transcription levels were consistently very low. However, correlations were observed between S100A2 and S100A7 (R = 0.48), S100A11 (R = 0.54), S100A14 (R = 0.50), S10016 (R = 0.61) and S100P (R = 0.85), when comparing transcript levels from the tumor array (Table 5).

### 3.2. S100A4

In cancer cell lines, the levels of S100A4 transcript were on average higher (mean Ct = 5.31 +/− 2.99) than those of other S100s, with large variations being observed between cell lines. AsPC-1 showed the highest level (Ct = 1.86 +/− 0.26), and CFPAC-1 showed the lowest transcript level (Ct = 10.13 +/− 0.73). Large variations in S100A4 levels in different pancreatic cancer cell lines have previously been reported [16,19,20]. An intermediate level of S100A4 transcript was observed in the HPNE control cell line (Ct = 3.62 +/− 0.75) (Table 3).

No correlation with statistical significance was observed between S100A4 and other S100s or HMGB1 in the cell lines (Table 4). However, statistically significant correlations were observed between S100A4 and other S100s in the tumor array (Table 5). Very strong correlations were observed between S100A4 and S100A9 (R = 0.78), and S100A10 (R = 0.78). Strong correlations were observed between S100A4 and S100A8 (R = 0.66), S100A12 (R = 0.64), S100A14 (R = 0.55), S100A16 (R = 0.60) and HMGB1 (R = 0.50). Moderate correlations were observed between S100A4 and S100A11 (R = 0.46).

### 3.3. S100A6

The transcript levels of S100A6 were overall very low in all cell lines (mean Ct = 12.1 +/− 0.83), with very little variation between cell lines. AsPC1 showed the highest level (Ct = 11.04 +/− 1.75) and CFPAC-1 the lowest level (Ct = 13.09 +/− 0.21). Interestingly, the immortalized HPNE cells show higher levels of S100A6 transcript (Ct = 7.93 +/− 0.31) than any of the cancer cell lines (Table 3).

No correlation was observed between the transcript levels of S100A6 and other S100s or HMGB1 in cells. However, in the pancreatic tumor array, correlations were observed between S100A6 and S100A11 (R = 0.49) and S100A14 (R = 0.45).

### 3.4. S100A7

Overall, all cell lines showed low levels (mean Ct = 14.19 +/− 1.53) of S100A7 transcript with little variations among the eight cell lines. CFPAC-1 presented the highest level (Ct = 12.12 +/− 0.21) and AsPC-1 the lowest (Ct = 15.9 +/− 0.53). The highest levels of S100A7 transcript were observed with the immortalized HPNE control cells (Ct = 5.93 +/− 1.64). Our results agree with those of Liu et al., who reported lower levels of S100A7 in a cohort of 45 pancreatic ductal adenocarcinomas when compared to adjacent non-tumor tissues [21].

No statistically significant correlation was observed between S100A7 and the other S100s, or with HMGB1, in the eight cancer cell lines. However, in tumor tissues, correlations were observed between S100A7 and S100A14 (R = 0.47), and S100P (R = 0.56), suggesting crosstalk between these different S100s in tumors.

### 3.5. S100A8

All cancer cell lines showed low levels of S100A8 transcript (mean Ct = 12.79 +/− 1.16). The lowest level was observed in MiaPaCa-2 cells (Ct = 13.87 +/− 0.36) and the highest level in HPAF-2 cells (Ct = 10.85 +/− 1.63). No transcript was detected in the immortalized control HPNE cells.

In cells, correlations were observed between S100A8 and S100A9 (R = 0.82), and S100A8 and S100P (R = 0.91). In the tumor array, correlations were observed between S100A8 and S100A4 (R = 0.66), S100A9 (R = 0.62) and S100A10 (R = 0.46).

### 3.6. S100A9

All cell lines showed overall low levels of S100A9 transcript (mean Ct = 11.11 +/− 1.99), with the lowest level in MiaPaCa-2 (Ct = 13.20 +/− 0.36) and the highest in BxPC3 (Ct = 7.17 +/− 1.01). As observed with S100A8, no S100A9 transcript was detected in immortalized HPNE control cells.

In cells, correlations were only observed between S100A9 and S100A8 (R = 0.82). In the tumor array, correlations were observed between S100A9 and S100A8 (R = 0.62), S100A4 (R = 0.78), S100A10 (R = 0.51), S100A12 (R = 0.76), and HMGB1 (R = 0.80).

### 3.7. S100A10

In the cancer cell lines, the transcript levels of S100A10 were among the highest of all S100s, with a mean Ct = 4.15 +/− 0.88. The highest level was observed in AsPC-1 (Ct = 3.34 +/− 0.90) and the lowest in MiaPaCa-2 (Ct = 5.97 +/− 0.33). Overall, the lowest level was observed with the immortalized HPNE control cell line (Ct = 6.93 +/− 1.05) (Table 3). Our results agree with previous reports that showed lower levels of S100A10 in normal tissues than in cancerous tissues [22,23,24,25,26,27].

In cells, no statistically significant correlation was found between S100A10 and the other S100s or HMGB1. However, in tumors, correlations were observed between S100A10 and S100A4 (R = 0.78), S100A8 (R = 0.46), S100A9 (R = 0.51), S100A12 (R = 0.51), S100A16 (R = 0.48), and HMGB1 (R = 0.70).

### 3.8. S100A11

The transcript levels of S100A11 were very similar across all eight cell lines (mean Ct = 6.71 +/− 0.65), the lowest level was observed in MiaPaCa2 (Ct = 7.49 +/− 0.13) and the highest in BxPC3 (Ct = 5.38 +/− 1.29). The lowest level was noted in the control HPNE pancreatic cell line (Ct = 7.70 +/− 0.87).

A statistically significant correlation was found between S100A11 and S100P (R = 0.73), both in cell lines (Table 4) and in the tumor array (R = 0.62) (Table 5). In addition, correlations were also noted in the tumor array between S100A11 and S100A2 (R = 0.54), S100A4 (R = 0.46), S100A6 (R = 0.49), S100A13 (R = 0.67), S100A14 (R = 0.65), and S100A16 (R = 0.78).

### 3.9. S100A12

Overall, the levels of S100A12 transcript were lower than those of S100A11 in all cell lines (mean Ct = 9.01 +/− 1.22). The lowest level was found in Panc-1 cells (Ct = 10.37 +/− 0.05) and the highest in CFPAC-1 (Ct = 6.54 +/− 0.2). S100A12 was detected at a similar level in the control HPNE cells (Ct = 8.12 +/− 0.67) and in the cancer cell lines (Table 3), in agreement with the results of the meta-analysis by Li et al. [28].

No statistically significant correlation was found between S100A12 and the other S100s or HMGB1 in cells; however, correlations were observed between S100A12 and S100A4 (R = 0.64), S100A9 (R = 0.76), S100A10 (R = 0.51) and HMGB1 (R = 0.62) in the tumor array.

### 3.10. S100A13

The transcript levels of S100A13 were relatively high in all cell lines with the highest level observed in Hs766T cells (Ct = 5.27 +/− 0.01) and the lowest in AsPC1 (Ct = 8.33 +/− 0.11). The levels of S100A13 transcripts were higher in the control HPNE cell line (Ct = 4.78 +/− 1.33) than in the cancer cell lines (Table 3).

A statistically significant correlation was found between S100A13 and S100A2 (R = 0.82), in cell lines, but no correlation between S100A13 and S100A2 transcript levels was observed in tumors. However, a correlation was found between the transcript levels of S100A13 and S100A11 (R = 0.67), in the tumor array.

### 3.11. S100A14

Very low levels of S100A14 transcript were found in all cell lines examined, with the lowest being observed in MIAPaCa-2 (Ct = 21.26 +/− 0.53) and the highest in BxPC3 (Ct = 8.96 +/− 1.14). No S100A14 transcript could be detected in the HPNE cell line (Table 3).

In the cell lines, no statistically significant correlation was found between S100A14 transcripts and the other S100s, or HMGB1. However, in the tumor array, positive correlations were observed between S100A14 and S100A2 (R = 0.50), S100A4 (R = 0.55), S100A6 (R = 0.45), S100A7 (R = 0.47), S100A11 (R = 0.65), S100A16 (R = 0.57) and S100P (R = 0.66).

### 3.12. S100A16

S100A16 transcript levels were relatively low in all cell lines, with the lowest level observed in Panc-1 cells (Ct = 9.27 +/− 0.23), and the highest ion AsPC1 cells (Ct = 6.25 +/− 1.02). The lowest levels were observed in the control HPNE cell line (Ct = 10.12 +/− 0.33) (Table 3).

As observed with S100A14, no statistically significant correlation was found between S100A16 and other S100s or HMGB1 in cells. However, in tumors, positive correlations were found between S100A16 and S100A2 (R = 0.61), S100A4 (R = 0.60), S100A10 (R = 0.48), S100A11 (R = 0.78), S100A14 (R = 0.57) and S100P (R = 0.54).

### 3.13. S100P

Very large variations in S100P transcript levels were observed between cell lines, with the lowest level observed in MIAPaCa-2 (Ct = 12.22 +/− 1.48) and the highest in BxPC3 (Ct = 4.18 +/− 0.18). S100P transcripts were not detected in HPNE control cells (Table 3).

In the cancer cell lines, statistically significant correlations were found between S100P and S100A8 (R = 0.91), and S100A11 (R = 0.73). A correlation between S100P and S100A11 was also observed in tumors (R = 0.62). In addition, positive correlations were found in tumors between S100P and S100A2 (R = 0.85), S100A7 (R = 0.56), S100A14 (R = 0.66) and S100A16 (R = 0.54).

### 3.14. HMGB1

HMGB1 levels were very similar in all cell lines, with the lowest level in CFPAC-1 (Ct = 8.67 +/− 0.32) and the highest in Capan-2 (Ct = 7.10 +/− 0.77). The lowest level of transcript was observed in the control HPNE cell line (Ct = 9.63 +/− 0.40) (Table 3).

A statistically significant positive correlation was observed between HMGB1 and S100P in cells (R = 0.71), and this correlation was also observed in tumors (R = 0.52). Additionally in tumors, correlations were found between HMGB1 and S100A4 (R = 0.50), S100A9 (R = 0.80), S100A10 (R = 0.70) and S100A12 (R = 0.62).

## 4. Discussion

For S100A2, S100A8, S100A9, S100A10, S100A11, S100A14, S100A16, S100P, and HMGB1, we observed higher transcript levels in the cancer cell lines than in the HPNE control cell lines. The higher levels of S100A2, S100A11, and S100A14 transcript in the eight cancer cell lines than in the HPNE control cell line suggest that these three S100s might modulate the behavior of the cancer cells. S100A2, S100A11, and S100A14 are three S100s with complex roles in cancer, as they have been described as either tumor suppressors or tumor promoters. For instance, S100A2 functions either as a tumor promoter or tumor suppressor, depending on the type of cancer [7,9,29]. Even within a specific type of cancer, such as pancreatic cancer, both tumor suppressor and promoter effects of S100A2 have been observed. In one study, high levels of S100A2 in pancreatic tumors were found to correlate with cancer progression and poor prognosis of patients [30,31]. However, in a different study, high levels of S100A2 correlated with high overall survival in pancreatic cancer patients treated with adjuvant therapy [32]. These conflicting reports clearly suggest that the role of S100A2 in pancreatic cancer is complex and appears to be dependent on yet unknown molecular characteristics of individual tumors.

Like S100A2, S100A11 is described to function as either a tumor suppressor, such as in bladder cancer, or a tumor promoter, such as in pancreatic cancer [7,9,29,33,34]. In pancreatic cancer, the levels of S100A11 were shown to increase during the early stages but decreased during the advanced stages of pancreatic cancer [35]. However, in patients, high expression levels of S100A11 in pancreatic ductal adenocarcinoma have been associated with an unfavorable prognosis for patients who had undergone surgical resection [36]. An unfavorable role of S100A11 in pancreatic cancer is also supported by a recent study in human Panc-1 pancreatic cancer cells, where the overexpression of S100A11 led to increased cell proliferation [37].

Similarly to S100A2 and S100A11, S100A14 appears to have a complex role in cancer, showing overexpression in breast, liver and cervical cancer, but downregulation in other cancers such as rectal, kidney, colon and esophageal cancers [38]. Chen et al. also showed that the overexpression of S100A14 in cancer cells could either induce or inhibit cell invasion, depending on the p53 status, as follows: increased cell invasion was observed in the p53 positive HT1080 fibrosarcoma and MCF7 breast cancer cell lines whereas a decrease in cell invasion was observed in the p53 negative non-small cell lung carcinoma cell line [39]. In the context of pancreatic cancer, Al-Ismaeel et al. showed that S100A14 repressed cell migration in BxPC3 cells, which carries p53 mutations [40].

The absence of detectable transcripts for S100A8 and S100A9 in HPNE cells was anticipated because these two S100s are mainly expressed in immune cells, such as monocytes and neutrophils [41]. For this reason, S100A8 and S100A9 can be found in tumor tissues that are infiltrated with immune cells, where they act as chemo-attractants for other immune cells and can promote an inflammatory tumor micro-environment [42]. Our observation that S100A8 and S100A9 transcripts were detected in the eight cancer cell lines, and not in the HPNE control cell line, suggests that these two S100s also act directly on pancreatic cancer cells. Pancreatic cancer cells might be stimulated by these S100s in an autocrine or paracrine manner. In support of this hypothesis, S100A8 has been shown to stimulate the migration of pancreatic cancer cells in vitro [43,44]. In addition, both S100A8 and S100A9 can be secreted in the extracellular environment and are RAGE ligands. Following their transcription, translation, and secretion, S100A8 and S100A9 might interact with RAGE, resulting in amplified RAGE signaling and increased tumor growth [41,45].

For S100A10, our observations agree with the current literature describing the role of this S100 in promoting the development of pancreatic tumors. S100A10 is upregulated in pancreatic cancer and contributes to cell proliferation, migration, invasion, and tumor growth [22,23,24,25,26,27,46]. S100A10 is also being considered as a biomarker in pancreatic ductal adenocarcinoma [26]. S100A10 could act in a paracrine or autocrine manner on pancreatic cancer cells.

S100A16 is one of the latest S100 family members identified and characterized [47]. Although several studies have shown that S100A16 participates in cancer progression in several cancers such as prostate, breast, colon, and lung cancer, not much is known on the role of S100A16 in pancreatic cancer [48,49,50,51,52]. A recent Oncomine database analysis revealed higher expression levels of S100A16 in human pancreatic cancer tissues than in normal control tissues, and that the prognosis of patients with high levels of S100A16 was less favorable compared to that of patients with lower levels of S100A16 [53]. In our study, the lower level of S100A16 transcript observed in the HPNE control cell line compared to the eight cancer cell lines, is in agreement with the Oncomine data analysis.

Our observations with S100P, where transcripts could only be detected in the cancer cell lines and not in the HPNE control cell line, are also in agreement with many studies that demonstrated the involvement of this S100 in the progression of pancreatic cancer [7,54]. Because of its high expression in pancreatic tumors, S100P is clinically used as a diagnostic marker of pancreatic ductal adenocarcinoma [55,56]. In pancreatic cancer cells, S100P stimulates cell proliferation and invasion through the interaction with RAGE [11,13,54,57,58,59].

HMGB1 was also found at lower levels in the HPNE control cells than in the cancer cell lines. The role of HMGB1 is complex with different cellular functions depending on the cellular localization [5]. In the nucleus, HMGB1 regulates gene transcription by binding to and stabilizing DNA, and has a tumor suppressor effect [4]. When present in the cytoplasm, HMGB1 increases drug resistance by promoting autophagy [4,60,61]. Finally, HMGB1 can also act as an alarmin or damage-associated molecular pattern (DAMP) by interacting with RAGE or Toll-like receptors (TLR) when secreted into the tumor microenvironment [5,62,63]. Indeed, blockage of the HMGB1/RAGE axis has been shown to reduce tumor growth and metastasis [64].

Although for most S100s, and for HMGB1, the transcript levels were lower in the control HPNE cells than in the cancer cell lines, this was not the case for S100A4, S100A6, S100A7, S100A12, and S100A13. For S100A7, the lower transcript levels observed in the cancer cell lines than in the control cell line are in agreement with the study of Liu et al., who reported lower levels of S100A7 in a cohort of 45 pancreatic ductal adenocarcinomas when compared to adjacent non-tumor tissues [21]. These observations suggest that S100A7 plays the role of tumor suppressor in the early stages of pancreatic cancer. Interestingly, Liu et al. also reported higher levels of S100A7 in invasive and secondary pancreatic cancer tumors than in primary tumors [21], further supporting the complex role of S100A7 in pancreatic tumor development by switching between tumor suppressor and tumor promotor functions.

For S100A13, based on the current literature, we anticipated observing lower levels of S100A13 in HPNE cells than in cancer cells. Indeed, a recent metadata analysis using Oncomine and GEPIA found significantly higher levels of S100A13 transcripts in pancreatic tumors than in control tissues [31]. Higher levels of S100A13 have also been found in melanoma and papillary thyroid carcinoma samples compared to normal samples [65,66]. However, it is important to note that the HPNE control cell line was established from pancreatic epithelial cells immortalized after transfection with human telomerase (hTERT). In hTERT-HPNE cells, senescence is reduced, and this could affect S100A13 transcription levels. Indeed, it was recently shown that S1000A13 promotes cellular senescence by facilitating the translocation of IL-1 alpha to the cell surface [67]. The deregulated senescence in HPNE cells could thus affect the regulation of S100A13 transcription in cells.

In our study, the transcripts of both S100A4 and S100A6 were found at a high level in the HPNE cells when compared to the pancreatic cancer cells (Table 3). As described with S100A13, S100A4 was recently shown to participate in cellular senescence by promoting the translocation of the p52 subunit of the nuclear factor kB (NF-kB) to the nucleus [68]. The role of S100A6 in the senescence of NIH 3T3 cells has also been previously reported [69]. As a result, the immortalization of HPNE cells through transfection with human telomerase could affect the levels of S100A4 and S100A6 in these cells as well.

The higher transcript levels of S100A12 observed in HPNE cells than in the cancer cells could also be indirectly caused by changes in the senescence abilities of these cells. Indeed, S100A12 was recently identified as 1 of the 20 upregulated genes of a senescence associated secretory phenotype (SASP) cluster [70]. Therefore, as indicated above for S100A4, S100A6 and S100A13, changes in the senescence properties of HPNE cells could affect the transcriptional regulation of S100A12.

The difference in transcript levels among the eight cancer cell lines was the smallest for HMGB1, with a Ct of 1.59, corresponding to a 3-fold difference among cell lines. S100A6, S100A10 and S100A11 showed between 4-fold and 8-fold differences in transcript levels among the cell lines. S100A7, S100A8, S100A12 and S100A16 showed larger differences with 8-to-16-fold differences among cell lines. Finally, S100s that showed the largest difference among cell lines were S100A9 (65-fold), S100A4 (308-fold), S100A2 (867-fold), S100P (324-fold) and S100A14 (5000-fold). It is reasonable to hypothesize that the observed variations in the transcript levels among the cell lines would also be observed at the protein levels; however, additional experiments that are beyond the scope of this study would be necessary to support this hypothesis. It is also important to note that our study only gives a snapshot of the levels of S100 and HMGB1 transcripts in the cancer cells in standard cell culture growth conditions and that cancer cells’ growth conditions are very different in the tumor environment. In our standard cell culture growth conditions, we only observed the following few correlations in transcript levels between S100s and HMGB1: correlations were observed between S100A2 and S100A13, S100A8 and S100A9, S100A8 and S100P, S100A11 and S100P and HMGB1 and S100P. On the other hand, in the context of the tumor, we observed 33 distinct positive correlations.

S100s have the following complex functions in cells: they can act intracellularly by activating different target proteins, mostly in a calcium-dependent manner, resulting in multiple cellular activities [7]. For instance, S100A4 can interact with multiple intracellular proteins (non-muscle myosin heavy chain (NMMHC) IIA, tropomyosin, and actin) that can result in changes in cell migration, explaining the role of S100A4 as promoting cancer metastasis [71]. S100A6 can interact with target proteins that lead to the ubiquitination and then degradation of b-catenin, therefore influencing the fate of the cells [72]. S100A10 in complex with annexin II assists trafficking of several membrane proteins to the plasma membrane [73]. S100A11 assists in DNA-double strand break repairs by interacting with rad54B [74]. By interacting with ezrin/radixin/moesin, S100P can promote trans-endothelial migration of tumor cells, thereby favoring metastasis [75]. As described above, interactions between S100s and their intracellular targets affect several aspects of tumorigenesis. In addition, many S100 proteins and HMGB1 act as alarmins as follows: they can be secreted outside the cells and act on neighboring cells, where they possess cytokine-like effects [8]. Many receptors for S100s and HMGB1 have been identified. They include the RAGE receptor, Toll-like receptor 4 (TLR-4), scavenger receptors, and heparan sulfate proteoglycans [7]. One limitation of our study is that we do not have information on the protein levels of the S100s and HMGB1 in the eight cell lines and in the tumor array. Here again, additional studies that are beyond the scope of the manuscript would be necessary to identify the alarmins secreted in the tumor micro-environment.

Our observation that a larger number of correlations was observed between the transcript levels of S100s and HMGB1 in the tumor array than in cells suggests that the tumor microenvironment provides complex transcriptional regulation mechanisms for S100s and HMGB1. It also suggests that many S100s and HMGB1 act in concert in pancreatic tumors. The upregulation of multiple S100s and HMGB1 in tumors could be a means for tumors to upregulate multiple S100-dependent pathways simultaneously.

As many of the S100s and HMGB1 are ligands of RAGE, it is reasonable to consider RAGE as one of the receptors responsible for the concerted upregulation of S100s and HMGB1 in tumors [76,77,78]. Indeed, in the context of melanoma xenograft tumors, we showed that overexpression of RAGE resulted in the upregulation of S100A2, S100A4, S100A6, and S100A10, both at the transcript and protein levels [79]. In a different study, using an in vivo model of murine pancreatic cancer, we showed that blocking the activation of RAGE using a RAGE-specific monoclonal antibody significantly reduced the protein levels of HMGB1 in tumors, suggesting that RAGE regulated the expression of HMGB1 in pancreatic cancer [60]. How can RAGE activation lead to the upregulation of its ligands? One possibility is through the activation of the hypoxia-inducible factor 1a (HIF-1a). Indeed, RAGE can signal through the RAF/MEK/ERK and the PI3K/Akt pathways, both resulting in the activation of NF-kB and HIF-1a in cells [80]. Because the promoter regions of many S100 genes contain a hypoxia responsive element (HRE), HIF-1a can upregulate their transcription. Indeed, HREs have been identified in the promoter regions of S100B [81], S100A2 [82], S100A4 [83], S100A8 and S100A9 [84]. Interestingly, HMGB1 has been shown to be secreted at high levels in hypoxic conditions in glioblastomas [85] and melanomas [86], resulting in tumor growth and metastasis. Because tumors contain not only cancer cells but also non-cancerous cells, such as immune cells, endothelial cells, and fibroblasts, they are the site of complex networks of signaling pathways [87]. To add to the complexity of signaling in tumors, RAGE is not only expressed in cancer cells but also in many other cells of the tumor microenvironment, such as immune cells [88], endothelial cells [89], and fibroblasts [90]. Therefore, the observed correlations at the transcript level of the S100s and HMGB1 described in this manuscript could be the result of complex transcriptional regulation orchestrated by RAGE in the different cells of the tumor micro environment.

## 5. Conclusions

Overall, we observed only the following five statistically significant correlations between S100s and S100/HMGB1 in cells: correlations were observed between S100A2 and S100A13, S100A8 and S100A9, S100P and S100A8 and S100A11, and S100P and HMGB1. However, when looking at the data from the tumor array, we observed 33 statistically significant correlations between S100s and S100/HMGB1. These positive correlations suggest similar mechanisms of transcriptional regulation between these genes and that many S100 family members act in concert in pancreatic tumors. This difference in data between cells and tumors probably reflects the complexity of tumors. Indeed, tumors consist not only of cancer cells but also non-cancerous cells, such as fibroblasts, immune cells, and endothelial cells. All these cells communicate with each other, and many of them express several S100 proteins. The transcription of S100s and HMGB1 in tumors is a very well-orchestrated process that integrates multiple signals from all cells present in the tumor.

## Figures and Tables

**Table 1:** Phenotype and genotype of the eight pancreatic cancer cell lines used in this study. Data taken from [18]. WT: Wild type; HD: Homozygous deletion. Note that Deer et al. reported conflicting results in the SMAD4 mutation status in AsPC-1 cell lines among different reports, and suggested that additional genetic alterations might have occurred during routine culturing in different laboratory [18].

Cell Line	Origin	Histologic Differentiation	KRAS	TP53	P16	SMAD4
AsPC-1	Ascites	Poor	G12D	R273H HD	WT	Conflicting results reported. WT/HD exons 1-11/Mut exon 2
BxPC-3	Primary tumor	Moderate to poor	WT	Y220C	WT	HD exons 1-11
Capan-2	Primary tumor	Well	G12V	WT	WT	WT
CFPAC-1	Liver metastasis	Well	G12V	C242R	WT	HD
HPAF-2	Ascites	Well	G12D	P151S	20–25 Del	WT
Hs766T	Lymph node metastasis	Non described	WT	WT	WT	HD
MIA PaCa-2	Primary tumor	Poor	G12C	R248W	HD	WT
Panc-1	Primary tumor	Poor	G12D	R273H	HD	WT

**Table 2:** Sequences of the primers used in this study.

Primer Names	Primer Sequence (5′- > 3′)
Fwd_qPCR_S100A2	CACTACCTTCCACAAGTACT
Rev_qPCR_S100A2	GAAGTCATTGCACATGAC
Fwd_qPCR_S100A4	CAAGTACTCGGGCAAAGAGG
Rev_qPCR_S100A4	CTTCCTGGGCTGCTTATCTG
Fwd_qPCR_S100A6	GGACCGCTATAAGGCCAGTC
Rev_qPCR_S100A6	GGTCCAAGTCTTCCATCAGC
Fwd_qPCR_S100A7	ACGTGATGACAAGATTGACAAGC
Rev_qPCR_S100A7	GCGAGGTAATTTGTGCCCTTT
Fwd_qPCR_S100A8	TGATAAAGGGGAATTTCCATGCC
Rev_qPCR_S100A8	ACACTCGGTCTCTAGCAATTTCT
Fwd_qPCR_S100A9	GGTCATAGAACACATCATGGAGG
Rev_qPCR_S100A9	GGCCTGGCTTATGGTGGTG
Fwd_qPCR_S100A10	AAAGACCCTCTGGCTGTGG
Rev_qPCR_S100A10	AATCCTTCTATGGGGGAAGC
Fwd_qPCR_S100A11	CTGAGCGGTGCATCGAGTC
Rev_qPCR_S100A11	TGTGAAGGCAGCTAGTTCTGTA
Fwd_qPCR_S100A12	AGCATCTGGAGGGAATTGTCA
Rev_qPCR_S100A12	GCAATGGCTACCAGGGATATGAA
Fwd_qPCR_S100A13	ACCACCTTCTTCACCTTTGC
Rev_qPCR_S100A13	AGGCGGCTTTACTTCTTCCT
Fwd_qPCR_S100A14	CATGAGCCATCAGCTCCTCT
Rev_qPCR_S100A14	TTCTCTTCCAGGCCACAGTT
Fwd_qPCR_S100A16	ATGTCAGACTGCTACACGGAG
Rev_qPCR_S100A16	TTCTGGATGAGCTTATCCGCA
Fwd_qPCR_S100P	ATGACGGAACTAGAGACAGCC
Rev_qPCR_S100P	AGGAAGCCTGGTAGCTCCTT
Rev_qPCR_HMGB	GGTGCATTGGGATCCTTGAA
Fwd_qPCR_HMGB	GCTCAGAGAGGTGGAAGACCA
Fwd_qPCR_human bactin	CACCATTGGCAATGAGCGGTTC
Rev_qPCR_human-actin	AGGTCTTTGCGGATGTCCACGT

**Table 3:** Average Ct values (Ct_gene_ − Ct_actin_) for each S100 and for HMGB1 gene, in each of the 8 cancer cell lines and in the HPNE non-cancerous control cell line tested. The standard deviations are indicated below each mean value. The mean Ct and its corresponding standard deviation for each ligand across all 8 pancreatic cancer cell lines cell lines is also indicated. ND: not detectable.

Cell Line	A2	A4	A6	A7	A8	A9	A10	A11	A12	A13	A14	A16	P	HMGB1
AsPC-1	17.35 +/− 3.53	1.86 +/− 0.26	11.04 +/− 1.75	15.9 +/− 0.53	13.27 +/− 0.99	12.25 +/− 1.00	3.34 +/− 0.90	7.07 +/− 1.37	9.26 +/− 0.21	8.33 +/− 0.11	11.33 +/− 0.72	6.25 +/− 1.02	8.89 +/− 0.61	7.33 +/− 0.62
BxPC-3	9.17 +/− 1.78	6.99 +/− 1.18	12.89 +/− 0.81	13.17 +/− 0.30	10.95 +/− 0.50	7.17 +/− 1.01	4.28 +/− 0.68	5.38 +/− 1.29	8.50 +/− 0.17	5.96 +/− 0.01	8.95 +/− 1.14	7.63 +/− 0.44	4.18 +/− 0.18	7.25 +/− 0.18
Capan-2	17.1 +/− 0.79	5.97 +/− 0.32	11.82 +/− 0.42	14.45 +/− 0.26	13.05 +/− 0.61	12.08 +/− 0.41	3.68 +/− 0.57	6.47 +/− 0.09	9.51 +/− 0.08	6.52 +/− 0.01	11.07 +/− 0.88	8.37 +/− 0.16	7.65 +/− 0.25	7.10 +/− 0.77
CFPAC-1	17.88 +/− 1.06	10.13 +/− 0.73	13.09 +/− 0.54	12.12 +/− 0.21	12.79 +/− 1.37	9.84 +/− 0.13	4.79 +/− 0.62	7.22 +/− 0.25	6.54 +/− 0.2	7.31 +/− 0.03	12.28 +/− 1.02	7.80 +/− 0.48	10.8 +/− 0.17	8.67 +/− 0.32
HPAF-2	17.59 +/− 0.77	7.84 +/− 0.45	12.25 +/− 0.43	15.16 +/− 0.22	10.85 +/− 1.63	10.42 +/− 1.14	3.36 +/− 0.19	6.59 +/− 0.55	10.38 +/− 0.13	6.71 +/− 0.08	10.29 +/− 1.36	9.16 +/− 0.20	3.88 +/− 0.63	7.33 +/− 1.23
Hs766T	11.4 +/− 0.65	1.91 +/− 0.51	11.67 +/− 0.38	12.70 +/− 0.29	12.70 +/− 2.28	11.03 +/− 0.19	3.72 +/− 0.52	7.00 +/− 0.56	7.95 +/− 0.07	5.27 +/− 0.01	19.32 +/− 0.82	7.76 +/− 0.54	7.14 +/− 0.47	8.15 +/− 0.55
MIAPaCa-2	16.78 +/− 1.72	2.84 +/− 0.30	12.91 +/− 0.08	13.75 +/− 0.92	13.87 +/− 1.29	13.20 +/− 0.36	5.97 +/− 0.33	7.49 +/− 0.13	9.61 +/− 0.33	6.96 +/− 0.05	21.26 +/− 0.53	8.98 +/− 1.75	12.22 +/− 1.48	8.35 +/− 1.05
Panc-1	18.93 +/− 0.79	4.93 +/− 0.98	11.05 +/− 1.8	13.29 +/− 1.19	13.56 +/− 1.05	12.95 +/− 0.84	4.07 +/− 0.55	6.50 +/− 0.36	10.37 +/− 0.05	7.64 +/− 0.01	20.69 +/− 0.43	9.27 +/− 0.23	10.18 +/− 0.18	8.09 +/− 0.26
Mean Ct (Cancer cell lines)	15.78 +/− 3.45	5.31 +/− 2.99	12.1 +/− 0.83	14.19 +/− 1.53	12.79 +/− 1.16	11.11 +/− 1.99	4.15 +/− 0.88	6.71 +/− 0.65	9.01 +/− 1.22	6.84 +/− 0.90	14.40 +/− 5.10	8.15 +/− 1.01	8.11 +/− 3.01	7.78 +/− 0.59
HPNE	19.80 +/− 0.12	3.62 +/− 0.75	7.93 +/− 0.31	5.93 +/− 1.64	ND	ND	6.93 +/− 1.05	7.70 +/− 0.87	8.12 +/− 0.67	4.78 +/− 1.33	ND	10.12 +/− 0.33	ND	9.63 +/− 0.40

**Table 4:** Pearson’s correlation analysis of the Ct values (Ct_gene_ − Ct_actin_) between S100/S100 and S100/HMGB1 pairs in pancreatic cancer cell lines. The *p* value is indicated for each pair. When statistically significant (*p* < 0.05), the R factor is indicated in parenthesis below the *p* value. Statistically significant *p* values are indicated in bold. Red values indicate that R > 0.7.

Pairs	A2	A4	A6	A7	A8	A9	A10	A11	A12	A13	A14	A16	S100P	HMGB1
**A2**	1	0.74	0.51	0.41	0.21	0.059	0.94	0.159	0.39	**0.027** **(0.82)**	0.7	0.44	0.17	0.64
**A4**		1	0.14	0.44	0.19	0.14	0.92	0.44	0.46	0.98	0.17	0.53	0.57	0.91
**A6**			1	0.25	0.4	0.17	0.075	0.92	0.24	0.43	0.63	0.68	0.97	0.46
**A7**				1	0.88	0.43	0.22	0.94	0.07	0.26	0.38	0.64	0.55	0.051
**A8**					1	**0.01** **(0.82)**	0.33	0.07	0.94	0.27	0.064	0.98	**0.0015 (0.91)**	0.22
**A9**						1	0.76	0.068	0.22	0.24	0.084	0.53	0.071	0.6
**A10**							1	0.4	0.6	0.98	0.22	0.45	0.088	0.081
**A11**								1	0.71	0.4	0.19	0.98	**0.04** **(0.73)**	0.097
**A12**									1	0.55	0.7	0.21	0.72	0.22
**A13**										1	0.94	0.69	0.2	0.89
**A14**											1	0.29	0.088	0.07
**A16**												1	0.88	0.61
**S100P**													1	**0.047** **(0.71)**
**HMGB1**														1

**Table 5:** Pearson’s correlation analysis of the Ct values between S100/S100 and S100/HMGB1 pairs in pancreatic tumors. The *p* value is indicated for each pair. When statistically significant (*p* < 0.05), the R factor is indicated in parenthesis below the *p* value. Statistically significant *p* values are indicated in bold. Red values indicate that R > 0.7. Green values indicate that 0.5 < R < 0.7. Purple values indicate that R < 0.5.

Pairs	A2	A4	A6	A7	A8	A9	A10	A11	A12	A13	A14	A16	P	HMGB1
**A2**	1	0.61	0.47	**0.035** **(0.48)**	0.76	0.82	0.92	**0.017** **(0.54)**	0.24	0.052	**0.028** **(0.50)**	**0.005** **(0.61)**	**4.2 × 10^−6^** **(0.85)**	0.12
**A4**		1	0.17	0.29	**0.008** **(0.66)**	**6.5 × 10^−5^** **(0.78)**	**0.0006** **(0.78)**	**0.049** **(0.46)**	**0.003** **(0.64)**	0.14	**0.014** **(0.55)**	**0.006** **(0.60)**	0.66	**0.027** **(0.50)**
**A6**			1	0.18	0.32	0.78	0.30	**0.032** **(0.49)**	0.09	0.38	**0.049** **(0.45)**	0.13	0.10	0.62
**A7**				1	0.088	0.55	0.58	0.074	0.34	0.27	**0.047** **(0.47)**	0.18	**0.012** **(0.56)**	0.57
**A8**					1	**0.004** **(0.62)**	**0.047** **(0.46)**	0.29	0.16	0.35	0.12	0.35	0.50	0.12
**A9**						1	**0.0006** **(0.51)**	0.83	**0.0001** **(0.76)**	0.53	0.61	0.29	0.38	**3.3 × 10^−5^** **(0.80)**
**A10**							1	0.11	**0.025** **(0.51)**	0.31	0.21	**0.036** **(0.48)**	0.92	**0.0007** **(0.70)**
**A11**								1	0.99	**0.005** **(0.67)**	**0.003** **(0.65)**	**0.7 × 10^−5^** **(0.78)**	**0.005** **(0.62)**	0.22
**A12**									1	0.51	0.87	0.48	0.068	**0.004** **(0.62)**
**A13**										1	0.054	0.06	0.087	0.74
**A14**											1	**0.01** **(0.57)**	**0.002** **(0.66)**	0.72
**A16**												1	**0.016** **(0.54)**	0.78
**S100P**													1	**0.021** **(0.52)**
**HMG--B1**														1

## Data Availability

Data is contained within this article.
